# The Efficacy, Safety, and Persistence of Therapy after Non-Medical Switching from an Originator Adalimumab in Inflammatory Bowel Disease: Real-Life Experience from Two Tertiary Centres

**DOI:** 10.3390/ph17101319

**Published:** 2024-10-02

**Authors:** Teodora Spataru, Remus Popescu, Monica State, Mihai Pahomeanu, Bogdan Mateescu, Lucian Negreanu

**Affiliations:** 1 Gastroenterology 1 Department, University Hospital, Carol Davila University, 020021 București, Romania; teodora.spataru@drd.umfcd.ro (T.S.); mihai.pahomeanu@drd.umfcd.ro (M.P.); 2 Gastroenterology Department, Colentina Hospital, Carol Davila University, 020021 București, Romania; monicastate4@gmail.com (M.S.); bogmateescu@gmail.com (B.M.)

**Keywords:** inflammatory bowel disease, Adalimumab, biosimilars, switch, non-medical

## Abstract

During the last two decades, an increased number of molecules with multiple mechanisms of action have been approved for the treatment of inflammatory bowel disease (IBD), with a substantial increase in the costs related to therapy, which has become a concern for payers, regulators, and healthcare professionals. Biosimilars are biologic medical products that are highly structurally similar to their reference products; have no clinically meaningful differences in terms of immunogenicity, safety, or effectiveness; and are available at a lower price. **Materials and Methods:** This was an observational prospective study conducted in two IBD centres in Bucharest and included 53 patients, 27 male (M) and 26 female (F), diagnosed with IBD according to standard clinical, endoscopic, radiological, and histological criteria, who were non-medically switched at the indication of the National Insurance House to a biosimilar of Adalimumab. **Aims:** The aim was to determine the rates of clinical remission, adverse effects, and treatment persistence at one year. **Results:** No significant differences were found in terms of the faecal calprotectin (FC) and C-reactive protein (CRP) levels 6 and 12 months after changing from the originator biologic treatment to a biosimilar. Only one patient required a change in their biological treatment following the clinical and biological loss of response. The main adverse effect reported by the patients was pain at the injection site. Of the 53 patients, only 2 reported pain at the injection site, and 1 patient reported experiencing abdominal pain and rectal bleeding immediately after the switch, but no recurrence was observed clinically or endoscopically. **Conclusions**: This observational study is the first to be carried out in Romania that shows that, after a non-medical switch, biosimilars of Adalimumab are as efficient and safe as the originator Adalimumab in the clinical treatment of patients with IBD.

## 1. Introduction

After the first use of infliximab in Crohn’s Disease (CD) in 1995, the wide-scale introduction of anti-tumour necrosis factor alpha (anti-TNF alpha) medications revolutionised the treatment of inflammatory bowel diseases (IBDs) [1].

During the last two decades, an increased number of molecules have been approved for the treatment of IBD, with a substantial increase in the costs related to therapy. The problem of the increased costs and access to therapy has become a preoccupation of payers and regulators, as well as healthcare professionals.

There has been a shift in the costs from those relating to hospitalisation and surgery to costs related to biologic therapies. Anti-TNF use was the main cost driver, accounting for 64% and 31% of the total cost of treatments for Crohn’s Disease (CD) and Ulcerative Colitis (UC), respectively, in the Netherlands, in seven academic and seven general hospitals [2]. A review on biologic therapy access in CD in Eastern European countries concluded that there is a strong correlation between the wealth of a country and the number of patients treated with biologics [3].

Biosimilars are biologic medical products that are highly similar to their reference products, with no clinically meaningful differences in terms of immunogenicity, safety, or effectiveness. The introduction of biosimilars has been seen as a way to provide a lower-cost alternative to the originator anti-TNF molecules and hence to increase treatment access and availability for patients [3,4].

Their introduction in gastroenterology was based on extrapolation, a decision to extend the efficacy and safety data from an indication (usually from rheumatology) for which the biosimilar has been clinically tested to other indications for which the reference product is approved [5]. Initially, due mainly to extrapolation, gastroenterologists were reserved in terms of biosimilar use and adoption, as shown by an ECCO survey in 2013, which demonstrated that only a minority of IBD specialists were aware of and confident about the benefits and issues generated by the use of biosimilars. It took only three years to increase the confidence in biosimilar use in gastroenterology, with a new survey in 2016 demonstrating that the originator and biosimilar were considered interchangeable by 44.4% of responders, as compared with 6% in 2013 [6].

However, among patients with IBD, there still persists a certain lack of knowledge about and confidence in biosimilars. In an Italian survey study among patients with IBD, the great majority of patients (73.9%) did not know whether originators and biosimilars could be considered equivalent, or whether the efficacy and safety of biosimilars were lower than those of the originators [7]. The authors concluded that substantial efforts from scientific societies and IBD patients’ associations were required to overcome this issue [7].

Another important concern is the nocebo effect, which is defined as a negative effect of a medical treatment that is induced by patients’ expectations and is unrelated to the physiological action of the treatment itself. Offering the appropriate information about the concept of biosimilars and their advantages in terms of increasing accessibility to an effective treatment, but also discussing the possibility of the nocebo effect, is part of the education of the IBD patient when therapeutic options are presented. This approach can lead to good clinical results, as shown through a recent study involving 210 patients with IBD who were non-medically switched to a biosimilar, where, despite an increase in early nocebo complaints within the first 6 months after the switch, no significant changes were found in terms of the clinical efficacy, biomarkers, therapeutic drug level, or anti-drug antibodies [8].

According to many IBD specialists, switching from the originator molecule with patients who are in stable clinical remission is currently acceptable. This is supported by position papers from different national associations. This switch appears to be as safe and effective as treatment maintenance with the originator, with no increased risk of immunogenicity [9,10].

Thus, the decision to start therapy with a biosimilar (naïve patients or switch) needs to be integrated with physicians’ knowledge about the patients and their disease on a case-by-case basis, after extensive explanations and according to local policies and reimbursement rules.

### Aim

At the end of 2022, a decision of the Romanian National House of Insurance recommended the non-medical switch of patients treated with Adalimumab to a biosimilar of the drug. The directive recommended that at least 50% of the existing patients treated with the originator Adalimumab be switched in the following year.

We decided to evaluate a cohort of patients from two tertiary centres that underwent a non-medical switch from the originator Adalimumab to a biosimilar, investigating the efficacy, safety, and side effects of this and the persistence of the treatment.

## 2. Results

In our population, there were 42 patients with Crohn’s Disease (50% male and 50% female) and 11 with Ulcerative Colitis (54.54% male and 45.45% female) who were switched to a biosimilar formulation of Adalimumab.

All patients but one in our cohort maintained clinical and biological remission at six and twelve months after switching treatments, with minimal adverse reactions.

At the time of switching, all patients were considered to be in clinical remission by their treating physician, although ten of the Crohn’s Disease patients had FC values > 200 µg/g, eight had FC values between 200 and 50 µg/g, and twenty-four had FC values < 50 µg/g. Six months after switching to one of the three biosimilars, we observed good clinical and biological control of the disease, with the majority of patients having FC values < 50 µg/g.

The good clinical evolution and the decrease in the calprotectin levels might be related to the intensive follow-up and tight control approach and due to the extensive explanations regarding biosimilar therapy and clinical evolution at each visit, strategies we used to further prevent the nocebo effect from switching.

Only one patient required a change to another biological treatment (Ustekinumab) due to the loss of clinical and biological response, an increase in calprotectin levels to above 1000, and the detection of an active disease during colonoscopy (the presence of deep ulcers and signs of inflammation in the colon and the ileo-colonic anastomosis).

No treatment escalation was needed, and no steroids were used in the 12 months of follow-up.

During the treatment with the originator Adalimumab, among the 42 patients with Crohn’s Disease (CD), 13 had a CRP level > 5 mg/dL while 29 had a CRP level < 5 mg/dL. Six months after switching to a biosimilar, eight patients had a CRP level > 5 mg/dL, and the remaining thirty-four had a CRP level < 5 mg/dL. Another parameter monitored was the serum ferritin level. During treatment with Humira, nine patients had a ferritin value < 30 µg/L, nineteen had ferritin levels between 30 and 100 µg/L, and the remaining fourteen had a ferritin level > 100 µg/L. Six months after switching to one of the three biosimilars, six patients had a ferritin level < 30 µg/L, twenty-four patients had levels between 30 and 100 µg/L, and twelve patients had a ferritin level > 100 µg/L (Table 1).

Regarding the FC and CRP values in patients with Ulcerative Colitis, during the treatment with originator Adalimumab, three patients had FC values > 200 µg/g, three had FC values between 200 and 50 µg/g, and five had FC values < 50 µg/g. Additionally, two had a CRP value > 5 mg/dL, and the remaining nine had a CRP value < 5 mg/dL. Six months after the initial period, two UC patients had FC values > 200 µg/g, two had FC values between 50 and 200 µg/g, and the remaining seven had FC values < 50 µg/g. The CRP values did not change significantly after switching (Table 2).

The secondary objective was to evaluate the occurrence of side effects related to the biosimilar therapy. The main adverse effect reported by our patients was pain at the injection site. Before switching, the use of the new administration devices (pens) for the biosimilars was extensively explained to the patients using videos and sham models of the pens.

One patient encountered a problem with the pen at the second administration of Hukyndra when it failed to deliver the drug after lifting the pen too early from the skin surface. Of the 53 patients, only 2 reported pain at the injection site, but this was considered insignificant, as the patients had a BMI under 20 and also reported the same symptoms with the original product.

Regarding other adverse effects (AEs), only one patient reported abdominal pain and rectal bleeding after the initiation of a biosimilar therapy. The adverse effects appeared after the first administration, were short-lived, and disappeared after two weeks. No clinical, biological, or endoscopic flare was noticed in this patient.

## 3. Discussion

The advent of biosimilars has been a significant milestone in the treatment of IBD, significantly reducing the costs of advanced therapies and allowing their widespread use. Biosimilars are similar but not identical to the reference biologic and, although they may contain different substances not found in the original preparation, the studies conducted to date have demonstrated high efficacy, safety, and comparable immunogenicity profiles [11,12,13,14,15,16].

Switching from originator Adalimumab to an Adalimumab biosimilar, or even from one biosimilar to another, for reasons driven by third parties such as payers—in other words, a non-medical switch—seems safe and effective [11,12,13,14,15]. However, the decision for a non-medical switch is often seen as arbitrary by physicians [17].

When the recommendation of the National Insurance House to switch to an Adalimumab biosimilar was issued, many gastroenterologists were still reluctant.

We decided to present the existing treatment options to our patients and to extensively discuss the concept of the biosimilarity of the drugs, allocating more time for the consultation to minimise the possible side effects after the switch, including the nocebo effect. Offering the appropriate information about the biosimilar drugs, their effectiveness, and their side effect profile (comparable to the originator), with the advantage of increased accessibility, and discussing the possibility of the nocebo effect is part of the education of the IBD patient when therapeutic options are presented [11].

During the follow-up, only one patient required the discontinuation of the treatment due to secondary failure and switched to another biologic, no patients were optimised, and patients remained in clinical remission without corticosteroids.

These results are better or comparable to those in the current literature reports [18,19,20,21]; however, the small number of patients and the short duration of the study are elements of possible bias.

Good clinical evolution after the switch and a decrease in the calprotectin levels were seen in a study comparing 93 CD patients that switched from originator Adalimumab to the SB5 biosimilar (Imraldi) to 93 matched controls continuing the originator. Although no statistically significant changes in the serum CRP or FC between weeks 0 and 10 were observed within or between the cohorts, in the switch group, the calprotectin values decreased at week 10 compared to week 0, and in the originator group, there was a slight increase in the calprotectin values [22]. The cohort was followed for 52 weeks (with 54 patients remaining in each group), with no clinically meaningful differences in CRP values, FC values, or drug concentrations at the end of the study [23].

A multicentre real-life study, which included 193 patients with UC, showed a higher difference (although not statistically significant) in favour of the T2T strategy for the individual primary and secondary outcomes, with the median FC values dropping from the baseline through month 12 as a result of treatment escalation based on this biomarker [24].

A remission rate of 74.5% and a treatment persistency of 81.6% at 12 months were shown in an Italian cohort of 98 patients switching from originator Adalimumab to Adalimumab SB5 (Imraldi). A higher number of patients reported mild adverse events (AEs) after the switch, most commonly injection site pain (maybe because the SB5 formulation contained sodium citrate until 2023) [11,25].

In a study from Hungary, the subjective efficacy of switching to a biosimilar was proven in the case of Adalimumab, while a more reduced efficacy was experienced with the infliximab biosimilar. The perception of AEs was high and varied between biosimilars [26].

The results of a real-life retrospective study of non-medical switching in Italy, which also included patients in stable remission under ADA originator treatment, showed that all ADA biosimilars were effective and safe. Clinical remission was maintained in 124 out of 153 patients (81.0%) after a median follow-up of 12 months in patients that switched from the originator [27]. However, the Italian real-life experience with a rate of about 20% of patients losing remission, together with a significant rate of successfully switching back to the ADA originator, is a finding that requires careful evaluation in prospective studies to assess both medical and ethical implications. This finding is particularly relevant for UC patients, who seemed to have the worst performance when the ADA originator was replaced for non-medical reasons in this study [28].

Regarding the safety profile, among the 53 patients, 2 experienced adverse reactions, such as pain at the injection site; however, this was in line with the known side effects of Adalimumab. Switching to a biosimilar can also mean changing the delivery device and patient care process, which can cause anxiety for the patient. Educating patients is essential to ensuring their comfort with the use of new devices. Additionally, both the physician and the medical team must be familiar with the new types of Adalimumab biosimilar delivery devices [11]. Factors contributing to pain during the administration of a subcutaneous product include the product’s composition, the injection speed, viscosity, the injection angle, the injection site, and patient-related factors. The biosimilar pen devices used in our study have a needle thickness of 29G and a length of 4–8 mm, similar to the original Humira product [11].

Our study has several limitations. Being an observational study, data collection is challenging by nature, and some data may be missing from medical records. Another limitation is the small sample size, especially the relatively small number of UC patients. This can be explained by the preference for other biologic treatments (infliximab or Vedolizumab) among UC patients. In addition, the follow-up period of monitoring of only 12 months is a limitation, as we recognise that a longer follow-up might add insightful information on the efficacy and persistence of the treatment.

## 4. Materials and Methods

This is an observational, multicentric, and prospective study conducted in two IBD centres in Bucharest on non-medical switching from Adalimumab.

This study included 53 patients (27 male and 26 female) diagnosed with Ulcerative Colitis or Crohn’s Disease based on standard endoscopic, radiological, and histological criteria. All the patients were followed actively using a tight control approach [29]. The extent of the disease was determined using the Montreal classification [30], and its severity was determined using the Mayo score for the UC patients [31] and the Harvey Bradshaw Index (HBI) for the CD patients [32].

The patients included in this study had completed at least induction treatment with the originator Adalimumab biologic (Humira) and were considered to be in clinical remission by their treating physician. As the prescription of Adalimumab formulations is issued monthly by hospital physicians, the patients were progressively non-medically switched to one of the Adalimumab biosimilars based on the recommendation of the National Insurance House at the moment of their first or second visit to the hospital for the prescription.

The choice of the new treatment brand for Adalimumab was not based on any predetermined criteria, and the decision was made by the current physician after an extended discussion with the patient. The Adalimumab biosimilars included in this study were those available at that moment, namely, Hukyndra, Imraldi, and Hyrimoz (Table 3) [11]. The initial dose and timing of administration were not altered in any patient after the switch to the Adalimumab biosimilars. All patients used injector pens for the administration of the biological treatment, and they received 40 mg of Adalimumab subcutaneously every other week.

All the included patients who switched from the ADA originator to an ADA biosimilar were clinically assessed before switching to a biosimilar and then were assessed monthly for twelve months (the duration of the study).

The following data were collected: demographics, previous and current therapy, clinical scores, and adverse effects related to the therapy. We also assessed some biological markers (CRP, meaning calprotectin), IBD Disk score, and IBD daily life burden.

The primary objective was the maintenance of clinical remission after switching from the original Adalimumab treatment to the Adalimumab biosimilar. Remission was defined as when a patient did not need ADA optimisation or the addition of steroids or immunosuppressants.

The secondary objective was to evaluate the presence of adverse effects after switching treatments, as well as the persistence of the therapy at 6 and 12 months.

## 5. Conclusions

This study on the non-medical switch to an Adalimumab biosimilar by 53 patients with IBD confirmed the efficacy of the Adalimumab biosimilars in the management of IBD patients, with excellent results at six and twelve months of follow-up. Furthermore, it demonstrates that switching from the original product to a biosimilar is safe, cost-effective, and therapeutically effective. No differences were observed between the three biosimilars used in this study.

This observational study is the first analysis conducted in Romania to show that Adalimumab biosimilars are as effective as the originator Adalimumab in the clinical practice relating to IBD patients. Our results support the idea that the widespread use of biosimilars does not affect therapeutic efficacy and patient safety.

## Figures and Tables

**Table 1:** Patients with Crohn’s Disease.

Characteristics	Humira	Hukyndra	Imraldi	Hyrimoz
**Patients**	42	11 (26.19%)	13 (30.95%)	18 (42.85%)
**Mean Age (years)**	44.07	43.54	42.38	45.61
**Sex**				
**M**	50%	81.81%	53.84%	27.77%
**F**	50%	18.18%	46.15%	72.22%
**Mean duration of treatment in months**	39.83	12	12	12
**Montreal Classification: age at diagnosis**				
**A1**	6	1	2	3
**A2**	22	7	7	8
**A3**	14	3	4	7
**Montreal Classification: location of disease**				
**L1**	14	4	4	6
**L2**	9	1	2	6
**L3**	19	6	7	6
**L4**	0	0	0	0
**Montreal Classification: behaviour over time**				
**B1**	31	9	7	15
**B2**	5	1	4	1
**B3**	6	1	2	2
**Faecal calprotectin**				
**>200 μg/g**	10 (23.8%)	0 (0%)	0 (0%)	0 (0%)
**200–50 μg/g**	8 (19.04%)	0 (0%)	0 (0%)	2 (11.11%)
**<50 μg/g**	24 (57.14%)	11 (100%)	13 (100%)	16 (88.88%)
**CRP**				
**>5 mg/dL**	13 (30.95%)	0 (0%)	0 (0%)	8 (44.44%)
**<5 mg/dL**	29 (69.04%)	11 (100%)	13 (100%)	10 (55.55%)
**Hb**	14.10 g/dL	13.7 g/dL	13.4 g/dL	12.4 g/dL
**Ferritin mg/L**				
**<30**	9 (21.42%)	0 (0%)	4 (30.76%)	2 (11.11%)
**30–100**	19 (45.23%)	5 (45.45%)	6 (46.15%)	12 (66.66%)
**>100**	14 (33.33%)	6 (54.54%)	3 (23.07%)	4 (22.22%)

**Table 2:** Patients with Ulcerative Colitis.

Characteristics	Humira	Hukyndra	Imraldi	Hyrimoz
**Patients**	11	3 (27.27%)	3 (27.27%)	5 (45.45%)
**Mean Age (years)**	51.72	63	43	50.2
**Sex**				
**M**	54.54%	66.66%	66.66%	40%
**F**	45.45%	33.33%	33.33%	60%
**Faecal calprotectin**				
**>200 μg/g**	3 (27.27%)	0 (0%)	1 (33.33%)	1 (20%)
**200–50 μg/g**	3 (27.27%)	1 (33.33%)	1 (33.33%)	0 (0%)
**<50 μg/g**	5 (45.45%)	2 (66.66%)	1 (33.33%)	4 (80%)
**CRP**				
**>5 mg/dL**	2 (18.18%)	0 (0%)	0 (0%)	2 (40%)
**<5 mg/dL**	9 (81.81%)	3 (100%)	3 (100%)	3 (60%)
**Hb**	14.22 g/dL	14 g/dL	13.5 g/dL	14.3 g/dL
**Ferritin**				
**<30 yg/L**	2 (18.18%)	0 (0%)	1 (33.33%)	1 (20%)
**30–100 yg/L**	3 (27.27%)	1 (33.33%)	0 (0%)	2 (40%)
**>100 yg/L**	6 (54.54%)	2 (66.66%)	2 (66.66%)	2 (40%)

**Table 3:** Adalimumab formulations available for prescription in Romania.

Brand Name	Humira	Imraldi	Hyrimoz	Hukyndra
**Producer**	Abbvie	Biogen/Ewopharma	Sandoz	Stada
**Needle G syringe**	29	29	27	29
**Needle G pen**	29	29	27	29
**Volume (mL) for 40 mg dosing**	0.4	0.4	0.4	0.4
**Pre-filled syringe/pen**	40 mg, 80 mg/40 mg, 80 mg	40 mg/40 mg	40 mg, 80 mg/40 mg, 80 mg	40 mg, 80 mg/40 mg, 80 mg
**Citrate**	No	No—since 2023	No	No
**Latex**	No	No	No	No
**Shelf life at 25 °C in days**	14	31	21	14

## Data Availability

Data is contained within the article.
